# Building capacity for testing sterile insect technique against *Aedes*-borne diseases in the Pacific: a training workshop and launch of sterile insect technique trials against *Aedes aegypti* and arboviral diseases

**DOI:** 10.1186/s40249-024-01239-8

**Published:** 2024-10-11

**Authors:** Nicole Foley, Florence Fouque, Qingxia Zhong, Herve Bossin, Jeremy Bouyer, Raman Velayudhan, Randall Nett, Anna Drexler

**Affiliations:** 1 Arboviral Diseases Branch, Division of Vector-Borne Infectious Diseases, Centers for Disease Control and Prevention, Fort Collins, CO USA; 2 Research for Implementation Unit, The Special Programme for Research and Training in Tropical Diseases, World Health Organization, Geneva, Switzerland; 3 Insect Pest Control Subprogramme, Joint FAO/IAEA Centre of Nuclear Techniques in Food and Agriculture, Department of Nuclear Sciences and Applications, International Atomic Energy Agency, Vienna, Austria; 4 Medical Entomology Laboratory, UMR 241 SECOPOL, Institut Louis Malardé, BP 30, 98713 Papeete, Tahiti French Polynesia; 5 Department of Control of Neglected Tropical Diseases, WHO, Geneva, Switzerland

**Keywords:** Vector-borne diseases, *Aedes albopictus*, *Aedes aegypti*, Sterile insect technique, Mosquito, Mosquito control, Public health, Pacific

## Abstract

**Background:**

Vector-borne diseases cause morbidity and mortality globally. However, some areas are more impacted than others, especially with climate change. Controlling vectors remains the primary means to prevent these diseases, but new, more effective tools are needed. The World Health Organization (WHO) prioritized evaluating novel control methods, such as sterile insect technique (SIT) for control of *Aedes*-borne diseases. In response, a multiagency partnership between the U.S. Centers for Disease Control and Prevention (CDC), the Special Programme for Research and Training in Tropical Diseases (TDR), WHO, and the International Atomic Energy Agency (IAEA) supported the operational implementation and evaluation of SIT against *Aedes aegypti* and arboviral diseases in the Pacific through a consortium of regional partners (PAC-SIT Consortium).

**Main text:**

A workshop was held from 2 to 6 May 2023, during which PAC-SIT country participants, researchers, and stakeholders in SIT, scientific advisory committee members, and organizational partners came together to review the principles and components of SIT, share experiences, visit field sites and the SIT facility, and officially launch the PAC-SIT project. Working in groups focused on entomology, epidemiology, and community engagement, participants addressed challenges, priorities, and needs for SIT implementation.

**Conclusions:**

The PAC-SIT workshop brought together researchers and stakeholders engaged in evaluating SIT for arboviral diseases in the Pacific region and globally. This training workshop highlighted that many countries are actively engaged in building operational capacities and phased testing of SIT. The workshop identified a key need for robust larger-scale studies tied with epidemiological endpoints to provide evidence for the scalability and impact on mosquito-borne diseases.

## Background

Vector-borne diseases (VBDs) are responsible for over 700,000 deaths per year and 17% of the global burden of infectious diseases [1]. Epidemic-prone arboviral diseases in particular, such as dengue, chikungunya, and Zika, are a growing global public health threat. Rising temperatures, global travel and trade, and changes in precipitation patterns have expanded the range of *Aedes* species mosquitoes that transmit these viruses, causing more arboviral disease outbreaks and in previously unreported areas [2].

For Pacific island countries and territories (PICTs) who are at the forefront of climate and health risk due to their geography and development status, outbreaks of mosquito-borne diseases have increased in frequency and scale [3]. In the last decade, PICTs experienced 104 arboviral disease outbreaks across 19 island countries/territories [4]. In French Polynesia alone, a Zika outbreak during 2013–2014 that caused 28,000 cases (11% of the population) [5, 6] was followed by a chikungunya outbreak in 2014 that resulted in 66,000 cases (25% attack rate) [4].

To address the increasing global burden of these diseases, the World Health Organization’s (WHO) Global Vector Control Response (GVCR) 2017–2030 [1] calls for countries to act to reduce mortality, cases, and epidemics, including by operationalizing novel methods for vector control. New tools and strategies also align with U.S. commitments under the National Health Security Strategy to protect against global health threats, including emerging and re-emerging infectious diseases [7].

Sterile insect technique (SIT) is a method of insect control using area-wide releases of sterile male insects to reduce target insect populations [8]. This strategy is regularly used in agriculture [9], and is being developed for public health mosquito control [10]. After WHO released SIT testing guidance in 2020 [11], the Special Programme for Research and Training in Tropical Diseases (TDR) at WHO, Department of Neglected Tropical Diseases (NTD) at WHO, the International Atomic Energy Agency (IAEA), and the U.S. Centers for Disease Control and Prevention (CDC) began collaborating to evaluate SIT as a potential new tool for arboviral vector control, starting with selected PICTs due to capacity and logistic considerations. Led by Institut Louis Malardé (ILM), the Pacific SIT Consortium (PAC-SIT) brings together Pacific region collaborators around SIT for controlling *Ae. aegypti* and *Aedes*-borne diseases.

Here, we describe proceedings of a training workshop held in Tahiti, French Polynesia during May 2–6, 2023 that convened researchers and stakeholders in SIT testing, expert advisors, and organizational partners to launch the PAC-SIT project. The workshop focused on *Aedes*-SIT implementation utilizing the phased conditional approach. Working groups on entomology, epidemiology, and community engagement met to discuss operational challenges and enhance participants’ capacities to design, deploy, and evaluate SIT.

## Main text

### Overview of the workshop

**Fig. 1:**
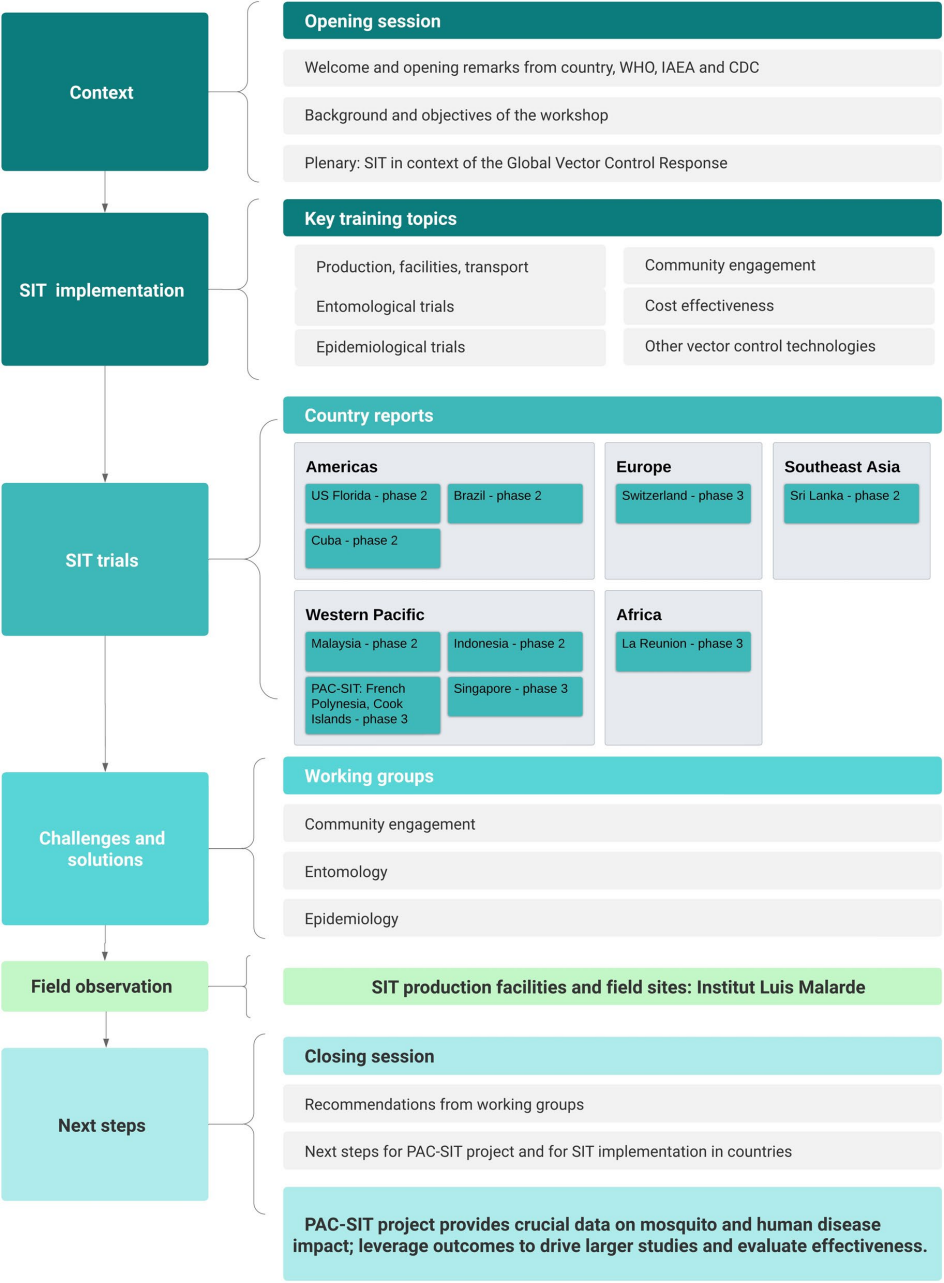
Logic diagram and overview of the PAC-SIT workshop structure. The workshop comprised topical sessions on various aspects of the sterile insect technique (SIT) implementation. The sessions provided contextual information, delved into specifics of SIT trials, discussed challenges and solutions, offered opportunities to visit field sites and production facilities, and concluded with recommendations for SIT development and the PAC-SIT project. *PAC-SIT* Pacific Islands Consortium for the Evaluation of *Aedes* SIT

The PAC-SIT workshop aimed to advance the implementation of SIT against *Aedes* species mosquitoes. The objectives of the meeting were to review principles and components of SIT, share experiences and case studies, learn through on-site observation; discuss challenges and officially launch the PAC-SIT project. The workshop began with welcoming addresses given by senior officials from the hosting institution, government of French Polynesia, CDC, IAEA, TDR and WHO, highlighting the importance of controlling arboviral diseases and potential of SIT as a tool for durable control. Workshop sessions dealt with SIT implementation, country and regional experiences, and working groups on community engagement, epidemiology, and entomological considerations for SIT trials (Fig. 1). Central to discussion were the PAC-SIT Phase 3 trials in French Polynesia and the Cook Islands to evaluate the impact of SIT on both mosquito populations and human disease. Participants were given the opportunity to visit the Medical Entomology Laboratory at Institut Louis Malardé, which specializes in operational research on innovative mosquito surveillance and control, and its production facilities for SIT- and *Wolbachia*- mosquitoes. Overall, the workshop provided a collaborative platform for researchers and stakeholders to share knowledge and insights on SIT implementation, contributing to sustainable health outcomes.

### SIT implementation

**Fig. 2:**
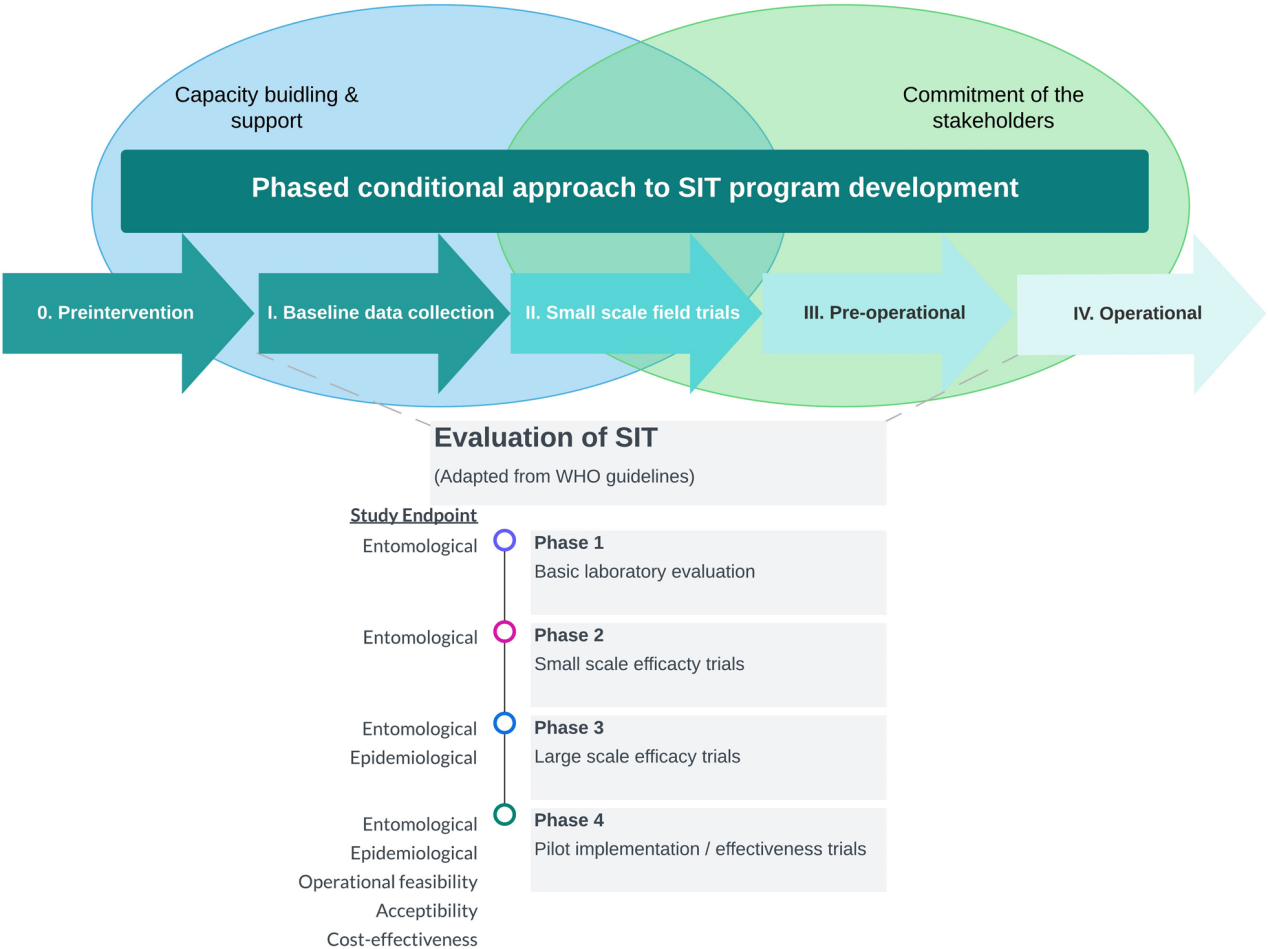
Sterile insect technique development and evaluation phases, adapted from references [11, 12]. *SIT* sterile insect technique; *WHO* World Health Organization

Members of the SIT Scientific Advisory Committee presented key SIT implementation topics, particularly relating to SIT evaluation and program development (Fig. 2). A major challenge in SIT and more generally of mosquito “Rear and Release” technologies is mass production of field competitive sterile male mosquitoes. Experts addressed building and running a mass production facility, irradiation processes, and quality control. Presentations highlighted best practices for trials to collect data on entomological impact, epidemiological trial principles and study designs, factors to consider for cost effectiveness analyses, and key approaches for community engagement, such as stakeholder mapping.

### SIT trials

Countries invited to the workshop presented SIT trials conducted in their regions of the world. The presentations were not exhaustive of all SIT field trials ongoing, instead presenting specific country experiences across various phases of SIT testing [11]. In addition to the trials described below, the PAC-SIT project plan and monitoring strategy was presented.

Despite a broad range of suppression trials conducted worldwide, robust evidence on the impact of SIT on disease epidemiology remains to be demonstrated. The PAC-SIT plan to implement SIT in two Pacific Islands to assess its effects on vector mosquito populations and human disease represents a significant step forward. This Phase 3 trial targets *Ae. aegypti* in Paea, Tahiti (population 13,000), French Polynesia and Aitutaki, Cook Islands (population 2200), both of which are experiencing dengue transmission this year. The study will use an integrated vector management (IVM) approach that combines community participation to remove larval containers around households with the release of sterile males. Mass production and irradiation of mosquitoes will be conducted by Institut Louis Malardé in Tahiti. ILM has been producing and releasing *Ae. polynesiensis* incompatible male mosquitoes since 2015. With advanced mosquito production and sterilization technology, their facility aims to produce up to 1,000,000 sterile *Ae. aegypti* males per week for the study. Weekly ground releases of chilled and compacted sterile adult males will be conducted, adjusting the releases based on field sterility levels inferred from ovitraps.

In both Paea and Aitutaki, study areas will span approximately 170 hectares per treatment, including buffer zones. The releases will occur over a 12-month implementation period with a release ratio of 10∶1 for sterile∶wild type males. Epidemiological metrics will include dengue seroprevalence and anti-mosquito saliva antibodies as a proxy for exposure to *Ae. aegypti* bites [13]. Entomological metrics will include egg and adult abundance, and bi-annual mark release and recapture (MRR) studies. Community engagement and acceptance was secured through stakeholder analysis and the development of a communication plan with culturally relevant communication materials. (Dr. Hervé Bossin and Dr. Françoise Mathieu-Daudé, Institut Louis Malardé, French Polynesia, unpublished).

#### African region (AFR) SIT Programs

Historically, SIT was successful against tsetse fly vectors in the African region. SIT elimination of tsetse from the island of Unguja, Zanzibar allowed farmers to increase production in the absence of animal trypanosomiasis [14, 15]. More recently, tsetse were eradicated from a 1000 km^2^ area around Dakar in Senegal [16], completely interrupting transmission of trypanosomiasis in cattle [17].

For mosquitoes, SIT against *Ae. albopictus* has been tested in La Réunion Island in the Indian Ocean [18], currently expanding to Phase 3 trials (pre-operational). The Phase 2 trials (small scale field trials) under controlled conditions carried out 2021–2022 were preceded by MRR studies in 2019 and 2021 and studies measuring egg counts, hatch rates, and adult abundance in response to SIT. The Phase 2 study included an immuno-epidemiological evaluation of *Ae. albopictus-*specific antibody responses as a measure of human-vector contact, and a knowledge, attitudes, and practices (KAP) survey to gauge community acceptability and support. The trial covered an area of 20 hectares, with weekly releases of 120,000–300,000 males, averaging 6000–15,000 males released per hectare. A Phase 3 study under natural conditions is planned and a new “boosted SIT” strategy that pairs SIT with Insect growth regulator (e.g. pyriproxyfen) autodissemination [19] will be tested (Dr. Frédéric Simard, Research Institute for Development, France, unpublished).

#### American region (AMR) SIT programs

All countries performing SIT trials in the American region are at Phase 2 and some will move to Phase 3 in the near future.

In Cuba, the Instituto Pedro Kouri and the national integrated mosquito management program conducted a Phase 2 trial against *Ae. aegypti* in Havana from February to September 2020 [20]. 40,000–80,000 sterile males were released twice a week, achieving up to 80% suppression. The entomological metrics were egg counts, hatch rates, induced sterility, and sterile male dispersion, survival, and competitiveness measured by MRR. SIT releases were consequently expanded across Havana in 2022 to evaluate the effectiveness of SIT to reduce mosquito abundance and dengue transmission (Dr. Rene Gato, Institute of Tropical Medicine Pedro Kourí [21]).

In Brazil (Recife and Juazeiro), a Phase 2 *Ae. aegypti* SIT trial was performed from October 2021 to January 2022. Efforts to optimize production, transport, and release procedures were presented. Before mosquito release, wild populations were suppressed with an integrated vector management (IVM) approach utilizing larvicides and mosquito-disseminated pyriproxyfen [22, 23]. Sterile males were released one to two times a week, with 0.5–0.7 million mosquitoes released per week, 5000–9000 males per hectare. Data collected included egg and adult abundance and hatch rates, and a MRR study in March 2021. Engagement of community leaders and local authorities, and public education is ongoing. The importance of applying SIT in an IVM framework was highlighted. The next steps for SIT implementation will be to expand to more areas in Brazil (Dr. Virginio Jair, Moscamed, Brazil, unpublished).

In the United States of America (USA), a Phase 2 *Ae. aegypti* trial on Captiva Island, Florida was implemented between 2020 and 2022. Sterile males were released across 300 hectares (3 km^2^) for both treatment and control zones, with releases exceeding 9 million mosquitoes in 2022. Entomological metrics included egg and adult abundance, and quarterly MRR studies [24]. Community outreach was conducted via town hall and local stakeholder meetings, and media outlets. Due to damage from Hurricane Ian in September 2022, the trial was relocated to Fort Myers, Florida (presented by Ms. Nicole Foley, CDC on behalf of Mrs. Rachel Morreale and Dr. David Hoel, Lee County Mosquito Control District, FL, unpublished).

#### European region (EUR) SIT programs

In Europe, where the establishment of invasive *Aedes* species in some areas is a concern, SIT could be an important tool for integrated mosquito management, advancing EU policy objectives to reduce biocide usage [Regulation (EU) No 528/2012] and proactively mitigating risk of outbreaks from introduced dengue, Zika, yellow fever and chikungunya. Several countries have initiated SIT programs, and most are in Phases 1 and 2 of development and testing. Notably, Italy and Switzerland have advanced to Phase 3, focusing on vector control to reduce nuisance biting rather than emphasizing epidemiological outcomes. This focus underscores regional priorities in these locales, where invasive *Aedes* species have established yet *Aedes*-borne diseases pose minimal public health threats compared to endemic regions like Asia or Latin America. In Ticino, Switzerland, a Phase 3 *Ae. albopictus* SIT trial occurred April through October 2023. Sterile males were produced in and transported from Italy (Centre Agricoltura Ambiente). 3000 males were released per hectare, with a total of 15,000 released per week. Entomological outcomes included fertility, dispersion, and survival. The importance of IVM in controlling local *Ae. albopictus* [25] was emphasized. (Dr. Elenora Flacio, University of Applied Sciences and Arts of Southern Switzerland, unpublished).

#### Southeast Asia region (SEAR) SIT programs

In Sri Lanka, a Phase 2 *Ae. albopictus* trial in Gampaha District has been completed [26]. The country utilizes IVM to control *Ae. albopictus*, however, insecticide resistance has hindered effective control, leading to the implementation of a SIT pilot during 2020–2021. Community engagement was conducted via door-to-door visits and focus groups. The trial area spanned 30 hectares with a target of 100,000 sterile males released per week. Entomological indicators included egg counts and hatch rates, adult abundance, and MRR [27] for sterile male dispersion and survival. Sri Lanka is developing production and irradiation capabilities to support a Phase 3 trial with expanded release areas (300 ha). (Dr. Anoja F. Dheerasinghe, Ministry of Health, Sri Lanka, unpublished).

#### Western Pacific (WPR) SIT programs

Indonesia started *Ae. aegypti* Phase 1 trials with laboratory activities in 2021 and moved to Phase 2 in 2022. The trial area covers 6.48 hectares for Phase 1 and 3.25 hectares for Phase 2, with 5881 males per hectare released in Phase 1 and 8970 males per hectare released in Phase 2. The trials monitored egg counts and hatch rates, and adult abundance. Community engagement with key stakeholders included a KAP survey to gauge community understanding of dengue and SIT. Plans for an epidemiological trial are underway (Mr. Hadian Iman Sasmita, National Research and Innovation Agency, Indonesia, unpublished).

In Malaysia, sterile males were released during 2019–2021 [28] and entomological data collected on larval counts and sterile male competitiveness, survival, and dispersal. The trial areas spanned from 4.55 to 8.41 hectares and between 1300–8000 males/hectare were released, totaling up to 2.2 million sterile males per study site. The country is now testing SIT under controlled field conditions (Phase 2), and presented staffing and training needs for the trials against *Ae. aegypti* in Malaka, Malaysia (Dr. Cheong Yoon Ling, Institute for Medical Research, Malaysia, unpublished).

Singapore is one of the countries most advanced in SIT testing, currently in Phase 3 for the SIT-incompatible insect technique (IIT) against *Ae. aegypti*. The SIT-IIT strategy involves releasing males that are both X-ray-sterilized and *Wolbachia*-infected to reduce mosquito populations [29]. For mosquito population surveillance, Singapore validated a Gravitrap *Aegypti* Index (GAI) [30]. National Environment Agency (Singapore) tested different mosquito release strategies ("rolling-carpet” and “targeted”) before starting this field study (NEA | Multi-site Field Study). The current Phase 3 study is a two-arm, cluster randomized trial that compares dengue case incidence between sites [31, 32]. Communities were surveyed for awareness, attitudes, and knowledge about SIT-IIT [33]. Engaging the community has been essential to the success of Singapore’s SIT-IIT program (Mr. Youming Ng, National Environment Agency, Singapore, unpublished).

### Challenges and solutions

Following the country SIT trials status updates, working groups were convened to discuss community engagement, epidemiology, and entomological considerations for SIT trials. An overview of priority challenges and solutions is given in Table 1.

**Table 1:** Summary outcomes of working group discussions on community engagement, entomology and epidemiology

Topic	Challenges	Solutions/needs
Community engagement	• Addressing misinformation and fake news, including social media impact • Sensitivity of communities to terminology like "Radioactive" in sterile insect technique (SIT) • The high costs associated with communication campaigns • Simplifying scientific information for lay audiences • Managing community expectations and explaining implementation stages (pilot vs scale-up) of SIT	• Include social scientists or communication experts in project teams • Develop guidelines for crafting and implementing a communication strategy, including for effective social media use • Develop standardized tools and resources [such as surveys, questionnaires, study designs, Information, education, and communication (IEC) material templates] to effectively assess community perceptions and monitor engagement • Create communication materials tailored for pilot and for operational phases of SIT for diverse audiences
Entomology	• Substantial startup costs • Increasing production and maintaining quality of mosquitoes • Managing variability due to blood supply, larval diet, water, and facility factors (e.g., ants) • Improving indicators to connect entomological data to epidemiology • High burden of surveillance during release phase • Lack of knowledge and data sharing among SIT trials • Adaptive release strategies based on real-time trap data	• Advocate for investment in SIT to reduce burden of high startup costs • Collaborate with medical entomology centers and develop production centers serving multiple programs to address cost and distribution challenges • Implement protocol improvements and automation technology to boost production and reduce staff workload; engage with industry to leverage advancements in manufacturing technology • Establish networks to facilitate knowledge sharing • Enhance production and quality through research on larval diet, artificial blood, managing genetic variation (local vs colony), adult irradiation and flight ability as fitness proxy • Advance data collection by developing real-time automated traps, population dynamic models, indicators/proxies for human-vector contact and age structure
Epidemiology	• During SIT trials, accounting for transmission that occurred outside of the trial area • Demonstrating SIT interventions directly contribute to outbreak prevention • Demonstrating effectiveness of SIT in areas with low disease incidence • Evaluating long-term success of SIT interventions • Determining number of trial sites and types of trials needed globally before declaring SIT effective • Aligning on a balanced approach to efficacy demonstration that ensures both scientific rigor and practical feasibility	• Guidance to interpret and contextualize negative associations (no disease transmission) • Review epidemiological indicators, including biomarkers as proxies, to identify the most effective metrics • Standardize data collection across SIT trials to facilitate meaningful meta-analyses • Guidance on duration of post-trial surveillance and number of trials in endemic and non-endemic locations required to confirm public health impact • Explore options for innovative and rigorous evaluation methods that can substitute for Randomized control trials (RCTs) with epidemiological outcomes

*SIT* sterile insect technique, *RCT* randomized control trials, *IEC* information education communication

The community engagement working group reviewed effective community engagement strategies applicable to SIT, stressing the importance of understanding the socio-cultural, historical, political, and economic context. They recommended involving a dedicated social scientist and communication focal point to support projects, along with the need for guidelines on developing, implementing, and evaluating SIT communication strategies and development of standardized tools like questionnaires and surveys. The group highlighted effective practices such as direct engagement methods (e.g., face-to-face interactions, door-to-door visits), participation in local events, and festivals to promote community involvement. They emphasized the value of participatory approaches that integrate community perspectives and transparent communication to build trust. Stakeholder mapping and collaboration with diverse stakeholders, including good will ambassadors and media partnerships, were also identified as crucial strategies to enhance community engagement and project success.

The epidemiology working group focused on addressing knowledge gaps and challenges in vector-borne disease epidemiology. They highlighted the importance and difficulties of implementing robust study designs like RCTs, which while providing important data, are costly and difficult to implement. The group emphasized serological markers and other surrogates for epidemiological impact, demonstrating impact in low disease incidence areas, and demonstrating epidemic prevention. They also discussed strategies to account for disease transmission outside of trial areas and debated the need for RCTs and alternative study designs to demonstrate efficacy, stressing the integration of scientific rigor with practical feasibility and standardized data collection.

The entomology working group discussed challenges, needs, and solutions across topics including SIT production, release strategies, and field evaluations. They noted high costs of production facility start-up, due in part to cost of irradiators, and the need for stable funding mechanisms and staffing. Distributed production (e.g., release centers that supply multiple programs) and increased translational research with industry towards more efficient equipment could reduce costs and allow more programs to access SIT. For field evaluations, egg density, sterility rate, and adult density are commonly measured, and adult indices should be prioritized. Challenges include connecting entomologic to epidemiologic outcomes, estimation of human-vector contact, and measures of mosquito population longevity/age structure. Other priority areas discussed included the need for more open knowledge and data sharing, and more advocacy for novel control tools like SIT.

## Conclusions

The PAC-SIT training workshop, held in Tahiti French Polynesia from May 2–6, 2023, served as a pivotal gathering to advance the implementation of sterile insect technique against *Aedes* spp. mosquitoes. This event brought together researchers, stakeholders, and expert advisors engaged in evaluating SIT against arboviral diseases in the Pacific region and globally, reflecting a collective effort to address the escalating global burden of these diseases. Participants shared experiences and country-specific challenges across SIT topics including production and logistics, entomology, epidemiology, and community engagement.

Five regions and ten countries shared their experiences with medium-to-large scale SIT testing. Central to this discussion were the PAC-SIT Phase 3 trials in French Polynesia and the Cook Islands, integrating SIT with community-based vector management to mitigate *Ae. aegypti* populations. These trials will employ comprehensive entomological and epidemiological metrics to evaluate effectiveness, including novel indicators like anti-mosquito saliva antibodies. Notably, workshop presentations highlighted the significant progress in SIT applications but did not cover all SIT trials conducted globally. According to a 2022 FAO/IAEA report to WHO VCAG, 42 trials are underway [34] with some using SIT alone and others combining SIT/IIT. One prominent example is that of Guangzhou, China, where an open-release field trial using the combined IIT-SIT approach nearly eliminated field populations of *Ae. albopictus* in the study sites [34] and further trials to optimize this approach are ongoing [35]. In the context of this workshop, the Phase 2 and 3 trial results presented demonstrate the potential of SIT strategies to significantly suppress mosquito populations and the importance of ongoing operational research for wider implementation.

The presentations and discussions emphasized the potential of SIT as a sustainable and scalable tool in integrated vector management strategies. However, challenges such as the high initial costs of production facilities and the complexity of demonstrating epidemiological impact were acknowledged. Despite progress in many countries’ respective SIT trials, information on the impact of this control technique on disease epidemiology is lacking. This gap highlights the need for robust larger-scale operational studies tied with epidemiological endpoints to provide proof-of-concept for scalability and impact on mosquito-borne diseases.

The PAC-SIT consortium project to evaluate SIT implementation in two Pacific Island locations aims to be the first study to collect both mosquito and human disease impact data for SIT-*Aedes*. This initiative sets an important precedent and underscores the need for similar initiatives in other settings to enhance the evidence base supporting SIT implementation. Future studies should focus on operational scalability and prioritize research efforts that bridge entomological findings with epidemiological outcomes. Additionally, expanding SIT networks and training opportunities is essential to foster collaboration and solve critical problems in the field.

In conclusion, the PAC-SIT workshop highlighted the active engagement, unique capacities and progress made by many countries using SIT for arboviral vectors. The collaborative efforts and insights shared will guide SIT development, leveraging sustained partnerships, innovative approaches, and evidence-based strategies to achieve sustainable public health outcomes.

## Data Availability

Not applicable.

## Abbreviations

WHO: World Health Organization
SIT: Sterile insect technique
TDR: Special Programme for Research and Training in Tropical Diseases
PAC-SIT: Pacific Islands Consortium for the Evaluation of *Aedes* SIT
VBDs: Vector-borne diseases
PICTs: Pacific island countries and territories
GVCR: Global Vector Control Response
ASPR: Administration for Strategic Preparedness and Response
NTD: Neglected Tropical Diseases
ILM: Institut Louis Malardé
CDC: Centers for Disease Control and Prevention
IAEA: International Atomic Energy Agency
MRR: Mark release recapture
IVM: Integrated vector management
KAP: Knowledge, attitudes, and practices
IIT: Incompatible insect technique
GAI: Gravitrap Aegypti Index
NEA: National Environment Agency
RCT: Randomized Controlled trial
